# The association of foot structure and footwear fit with disability in children and adolescents with Down syndrome

**DOI:** 10.1186/s13047-015-0062-0

**Published:** 2015-02-12

**Authors:** Polly QX Lim, Nora Shields, Nikolaos Nikolopoulos, Joanna T Barrett, Angela M Evans, Nicholas F Taylor, Shannon E Munteanu

**Affiliations:** 1 Discipline of Podiatry, College of Science, Health and Engineering, La Trobe University, Bundoora, Victoria 3086 Australia; 2 Discipline of Physiotherapy, College of Science, Health and Engineering, La Trobe University, Bundoora, Victoria 3086 Australia; 3 Lower Extremity and Gait Studies Program, La Trobe University, Bundoora, Victoria 3086 Australia

**Keywords:** Foot, Shoes, Flat foot, Foot deformities, Hallux valgus, Down syndrome

## Abstract

**Background:**

Foot deformity, flat feet, and the use of ill-fitting footwear are common in children and adolescents with Down syndrome (DS). The aim of this study was to determine whether these observations are associated with foot-specific disability in this group.

**Methods:**

A cross-sectional study design. Foot structure (foot posture determined using the Arch Index, presence of hallux valgus and lesser toe deformities) and footwear fit (determined by length and width percentage differences between the participant’s foot and footwear) were assessed in 50 participants with DS (22 females, 28 males) aged five to 18 with a mean (SD) age of 10.6 (3.9) years. Foot-specific disability was determined using the parent-reported Oxford Ankle Foot Questionnaire for Children (OxAFQ-C). Associations between foot structure and footwear fit with the four domains (Physical, School and play, Emotional and Footwear) of the OxAFQ-C were determined using multivariate regression modelling.

**Results:**

The mean (SD) Arch Index was 0.29 (0.08), and the prevalence of flat feet, hallux valgus and lesser toe deformities was 76%, 10% and 12% respectively. Few participants wore footwear that was too short (10%), but the use of footwear that was too narrow was common (58%). The presence of hallux valgus was significantly associated with increased disability for the OxAFQ-C School and play domain scores. The use of narrow-fitting footwear was significantly associated with increased levels of disability for the OxAFQ-C Physical, School and play, and Emotional domains. However, these variables only explained between 10% to 14% of the variance in the OxAFQ-C domain scores. There were no significant associations between foot structure and footwear fit with the OxAFQ-C Footwear domain scores.

**Conclusions:**

Flatter feet and lesser toe deformities are not associated with foot-specific disability in children and adolescents with DS. Hallux valgus is associated with foot-specific disability during school and play activities. Ill-fitting footwear (too narrow) is common and is associated with foot-specific disability. Further research is required to identify if the relationship between narrow-fitting footwear and foot-specific disability is causal, and to identify other factors associated with foot-specific disability in children and adolescents with DS.

## Background

Down syndrome (DS), also known as Trisomy 21, is the most prevalent chromosomal disorder and is caused by the presence of all or part of a third copy of chromosome 21 [1,2]. DS is primarily characterised by variable intellectual disability, distinct facial phenotype, short stature, generalised joint laxity and hypotonia [2,3]. Physical disability is an additional problem in people with DS and manifests as reduced physical fitness [4], reduced lower limb muscle strength [5], less functional gait patterns [6] and gait instability [7].

Musculoskeletal foot disorders are prevalent in individuals with DS [8-15]. In a population-based study of 197 young adults with DS aged 15 to 30 years, foot problems were reported to be the most prevalent muscle and bone condition, affecting nearly two-thirds of participants [12]. Of these participants, 66% reported that foot problems frequently affected their daily life [12]. Other studies have shown that children and adolescents with DS are also more likely to have foot conditions than their peers without DS [16-18]. There is evidence that children and adolescents with DS have flatter feet [16-18] and are more likely to have structural foot disorders such as hallux valgus [16]. Due to the presence of hallux valgus, footwear-fitting problems have been a concern in individuals with DS [11,17]. There is evidence to suggest that children with DS were more likely to wear ill-fitting footwear [17].

While it has been recognised that children with DS are more likely to have flatter feet, hallux valgus and footwear-fitting problems, no study has attempted to determine if there is a relationship between these characteristics with foot-specific disability in this group. Therefore, the aim of this study was to determine if there is an association between foot structure and footwear fit with the extent of disability related to foot problems in children and adolescents with DS.

## Methods

This cross-sectional study was approved by the Human Ethics Committee of La Trobe University, Australia (HEC13-035).

### Recruitment

**Figure 1:**
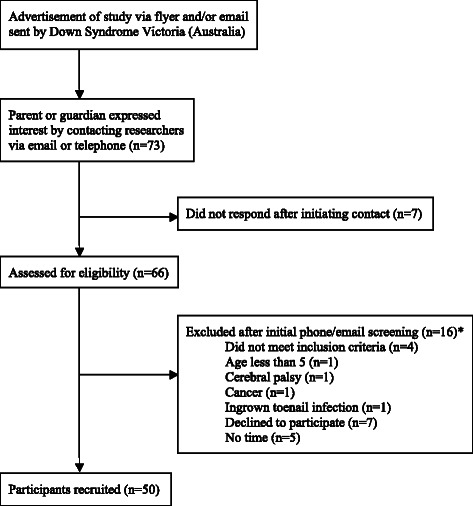
**Participant recruitment flowchart.** *Some volunteers were excluded for multiple reasons.

Children and adolescents with all genetic variants of DS aged five to 18 years were invited to participate in this study through email advertisements and flyers distributed to members of Down Syndrome Victoria. Participants were excluded if they had any previous lower limb surgery, were unable to walk without a supportive device such as a walker or brace, or had a concomitant medical condition or injury that could affect their physical function (e.g. neurological or inflammatory disorder). Fifty children and adolescents with DS aged five to 18 years were recruited. The participant recruitment process is shown in Figure 1.

### Data collection

From October 2013 to May 2014, assessments were conducted at a university health sciences clinic (La Trobe University, Melbourne, Victoria, Australia) by the same investigator (PQXL). Written informed consent was obtained from all parents or guardians prior to data collection. Where possible, consent was also obtained from participants aged 13 years and above. All of the participants’ parent or guardian completed a participant information questionnaire concerning the participants’ medical history and current medications prior to the assessment. The participants’ age, sex, height (cm), weight (kg), body mass index (kg/m^2^), and use of foot orthoses were documented.

### Assessment of foot structure

#### Foot posture

Foot posture was assessed using the Arch Index [19]. The Arch Index was calculated by obtaining a footprint using PressureStat^TM^ carbon paper (Bailey Instruments Ltd, UK) with the participant in a relaxed weight-bearing stance position. The weight-bearing length of the footprint (cm) was determined by measuring the length from the centre of the base of the heel to the centre of the front edge of the forefoot (excluding the toes). The front edge was determined as the front weight-bearing border of the forefoot marked on a line extending from the second digit in the transverse plane [20]. Areas of forefoot, midfoot, and rearfoot regions were then determined by dividing the length of the foot into equal thirds [20]. The surface area of each third of the weight-bearing regions was calculated using a graphics tablet and stylus (Wacom Technology, Vancouver, Canada) and an image analysis software Scion® (Scion Corporation, Chicago, Illinois, USA). The Arch Index was then determined as the ratio of area of middle third of the footprint to the entire footprint excluding the toes, with a higher ratio indicating a flatter foot [19]. This measure has been shown to exhibit excellent reproducibility in child and adolescent populations [21,22] and has been validated against radiographs of the foot [23]. There is evidence that assessment of the Arch Index can be affected by body composition [24,25]; this may be a concern as people with DS are more likely to be overweight [2,26,27]. However, recent work has shown that measures of Arch Index are not affected by body mass index in children and adolescents with DS [28].

#### Presence of hallux valgus and lesser toe deformities

The severity of hallux valgus was determined using the Manchester scale grading system [29]. This measure has good reproducibility (kappa coefficients = 0.80 to 0.89) [29,30] and has been validated against radiographic measurements [31]. The degree of deformity was graded on a scale of 0 to 3 (no deformity to mild, moderate and severe deformity) based on observation of the participant’s foot in a relaxed bipedal stance. Scores for Manchester scale of hallux valgus were dichotomised where a score of 0 or 1 was categorised as absent, and scores of 2 and 3 were categorised as present for hallux valgus [31]. Manchester scale grades of 2 (moderate deformity) and 3 (severe deformity) were chosen to represent the presence of hallux valgus as previous work by Garrow et al. [29] has shown that raters have little difficulty distinguishing between mild (score 1) and moderate (score 2) deformity. However, raters have difficulty distinguishing between no deformity (score 0) and mild deformity (score 1). In addition, previous work, using radiographic assessment as the gold standard, has shown that although there are significant differences in the mean hallux abductus angle between all Manchester scale grades, the total range of values overlaps for the ‘none’ and ‘mild’ deformity grades, indicating that differentiation between milder levels of deformity may not always be accurate [31]. Lesser toe deformities were documented by observing the presence of sagittal plane deformities (that is, claw, hammer or mallet toes) of the second to fifth digits while the participant was standing in a relaxed weight-bearing position. This assessment has good reproducibility as inter-tester reliability has previously been shown to have an absolute agreement of 85% [32].

### Footwear fit assessment

Footwear fit of the participants’ most regularly worn footwear was measured using the technique described previously [33]. The outline of the sole of each shoe was traced onto graph paper. A tracing of participant’s foot was performed while the participant stood in a relaxed bipedal position on the PressureStat^TM^ footprint mat. Footwear and foot tracings were then retraced using a computer graphics tablet and stylus (Wacom Technology, Vancouver, Canada) in conjunction with an image analysis software Scion® (Scion Corporation, Chicago, Illinois, USA). In cases where the upper of the footwear was wider than the sole, the boundary of the upper was used in the analysis [33]. The maximum length and width of the participant’s foot and footwear were documented in millimetres. This method has sound intra-rater reproducibility (ICCs > 0.99, coefficients of variation between 0.3 and 1.2%) [33]. The percentage difference between the foot and footwear dimensions was calculated for length and width measurements. Positive values indicate that the footwear dimension is larger than the corresponding foot dimension.

### Assessment of foot-specific disability

The disability associated with foot and ankle problems was assessed using the Oxford Ankle Foot Questionnaire for Children (OxAFQ-C) [34]. The 15-item questionnaire assesses the severity of foot and ankle problems across three domains of disability, (i) Physical (6 items), (ii) School and play (4 items), and (iii) Emotional (4 items). A final domain (item 15: Footwear) is not considered to be an assessment of disability [34], however it was included in our analysis as it is considered to reflect the concern of whether the participant’s foot or ankle stopped them from wearing the footwear they wanted to wear [34]. The scoring system assesses how frequently each issue (represented by an item in the questionnaire) is a problem. The response options to each item are on a 5-point scale rated from never (4), rarely (3), sometimes (2), very often (1) to always (0). Domain scores were calculated as the total of the scale item scores divided by the maximum for each domain (i.e. Physical 24, School and play 16, Emotional 16 and Footwear 4). Domain scores were then transformed to a percentage scale (0 to 100%) [34]. A lower score for a domain represents greater disability [34].

The OxAFQ-C is available in a child- or parent (proxy)-reported version. We administered the parent version of the OxAFQ-C because it was assumed that the child version would not be reflective of the participant’s response as the participants would be unable to provide responses given their level of intellectual disability and age (e.g. 5 years old). The OxAFQ-C has demonstrated good child–parent agreement for all OxAFQ-C domains (Physical ICC = 0.78; School and play ICC = 0.84; Emotional ICC = 0.73; and Footwear ICC = 0.65). The OxAFQ-C has also demonstrated both cross-sectional and longitudinal validity [34,35] as an assessment tool across a range of paediatric conditions of the foot and ankle [36].

### Sample size determination

Using G*power 3.1 software [37] for performing a sample size calculation, we estimated that we would need a minimum of 46 participants to detect a significant association between the foot structure and footwear fitting variables with each domain of the OxAFQ-C using linear regression models. This sample size was estimated using a *p* value = 0.05, power = 0.8, effect size (*R*^*2*^) = 0.15 for each model, and number of predictors = 4 (each foot structure and footwear fitting variable, with age, sex, body mass index).

### Statistical analysis

All analyses were performed using IBM SPSS Statistics for Windows, Version 22.0 (Armonk, NY: IBM Corp.) and *p* values < 0.05 were considered statistically significant. Data were assessed for normality and any non-normally distributed data were transformed using natural logarithm (Ln) where possible. In order to satisfy the independence assumption of statistical analysis, only measurements from the participant’s right foot and footwear were analysed [38].

We initially explored our data by analysing univariate associations (assumes that the response variable is influenced only by a single factor) between potentially associated variables with the domain scores of the OxAFQ-C using correlation statistics, independent samples *t*-tests, or Mann–Whitney U-tests (specific approach depended on the scaling and distribution of the data).

To determine the relationship between foot structure and footwear fit variables with the domains of the OxAFQ-C we used a hierarchical multivariate regression approach to determine the relationship after adjusting for age, sex and body mass index. This statistical approach allowed us to determine the relationship between potentially associated variables with the domain scores of the OxAFQ-C when controlling for confounding factors (i.e. age, sex and body mass index). In these analyses, footwear fit was dichotomised into two categories by transforming the percentage difference (between dimensions of the shoe and foot) into medians. The lower median represented poorer fitting footwear (i.e. footwear of insufficient length and width) and the upper median represented better footwear fit (i.e. footwear of appropriate length and width).

We used multivariate linear regression models for analyses where the Physical and School and play domains of the OxAFQ-C were the outcome variables. The fraction of variance independently explained by the foot structure or footwear fit variables was determined using the *R*^*2*^ change statistic and the associated *p* value. We used multivariate logistic regression models for the analyses where the Emotional and Footwear domains of the OxAFQ-C were the outcome variables as we were unable to transform these variables to be normally distributed. For each variable in the logistic regression models, odds ratios (OR) and 95% confidence intervals (CI) were determined (after adjusting for sex, age and body mass index). The fraction of variance independently explained by the foot structure or footwear fit variables was determined using the change in Nagelkerke *R*^*2*^ statistic and the associated *p* value [39].

## Results

Participant characteristics are shown in Table 1. Twenty-eight (56%) of participants were male, and the group had a mean (SD) age of 10.6 (3.9) years (range five to 18 years). Thirty-eight participants (76%) were classified as having flat feet, five (10%) had hallux valgus (all were classified as moderate severity) and six (12%) had lesser toe deformities. Five participants (10%) wore footwear shorter than their feet, and 29 (58%) wore footwear narrower than their feet. The mean (SD) of the OxAFQ-C Physical domain score was 72.8% (21.4), the OxAFQ-C School and play domain score was 84.9% (18.1), the OxAFQ-C Emotional domain score was 92.9% (13.8), and the OxAFQ-C Footwear domain score was 67.5% (3.9) (Table 1).

**Table 1:** **Participant characteristics**

Characteristic	Value	Range
General		
Age (years), mean (SD)^a^	10.6 (3.9)	5 to 18
Sex, n (%) male	28 (56)	N/A
Type of DS, n (%)		N/A
Trisomy 21/Translocation/Mosaic	44 (88)/5 (10)/1 (2)	
Any type of medication, n (%)	23 (46)	N/A
Height (cm), mean (SD)	131.9 (18.6)	96.0 to 164.5
Weight (kg), mean (SD)	39.6 (18.4)	16.3 to 85.1
Body mass index (kg/m^2^), mean (SD)		
Overall	21.5 (5.3)	13.8 to 34.9
Males	21.1 (4.9)	14.7 to 33.8
Females	22.1 (5.8)	13.8 to 34.9
Foot structure		
Arch Index, mean (SD)	0.29 (0.08)	0.01 to 0.39
Foot posture, n (%)^b^		
Flat/typical/high arch	38 (76)/6 (12)/6 (12)	
Hallux valgus, n (%)	5 (10)	N/A
Lesser toe deformity, n (%)	6 (12)	N/A
Footwear fit^c^		
Length (%)	9.0 (7.5)	−14.3 to 23.3
Width (%)	−4.5 (10.0)	−26.5 to 13.6
OxAFQ-C domain scores, mean (SD)		
Physical^d^ (%)	72.8 (21.4)	33.3 to 100.0
School and play^d^ (%)	84.9 (18.1)	31.3 to 100.0
Emotional^d^ (%)	92.9 (13.8)	43.8 to 100.0
Footwear (%)	67.5 (3.9)	0.0 to 100.0

^a^SD: Standard deviation.

^b^Based on the Arch Index, foot postures were classified as follows: Arch Index ≥ 0.26 (flat), 0.22 < Arch Index < 0.26 (typical), Arch Index ≤ 0.21 (high arch) [19].

^c^Percentage difference between shoe and foot dimensions. Positive values indicate shoe dimension greater than foot dimension. Measurements were based on *n* = 48 due to missing data.

^d^Parent-reported Oxford Ankle Foot Questionnaire for Children (OxAFQ-C) domain scores were based on *n* = 49 due to missing data.

### Univariate associations between participant characteristics, foot structure and footwear fit with OxAFQ-C domain scores

Pearson’s product moment correlations (*r*) and Spearman’s rank-order correlations (ρ) for the associations between participant characteristics (continuous scaled variables) and OxAFQ-C domain and item scores are shown in Table 2. The age, height and weight of participants were significantly inversely associated with the OxAFQ-C Emotional domain scores (ρ = −0.37, *p* = 0.009; ρ = −0.34, *p* = 0.016; ρ = −0.34, *p* = 0.018 respectively). Body mass index was significantly inversely associated with the OxAFQ-C School and play domain scores (*r* = −0.30; *p* = 0.039).

**Table 2:** **Univariate associations (correlations) between continuous scaled variables and OxAFQ-C domain scores**

Participant characteristics	Physical^a^		School and play^a^		Emotional^a^		Footwear	
	Univariate association^b^	*p*value	Univariate association^b^	*p*value	Univariate association^c^	*p*value	Univariate association^c^	*p*value
Age (years)	−0.155	0.289	−0.190	0.191	−0.370	0.009	0.020	0.889
Height (cm)	−0.192	0.186	−0.198	0.172	−0.342	0.016	−0.009	0.949
Weight (kg)	−0.274	0.056	−0.273	0.058	−0.338	0.018	−0.070	0.630
Body mass index (kg/m^2^)	−0.278	0.053	−0.295	0.039	−0.272	0.059	−0.199	0.166
Arch Index	−0.236	0.102	−0.245	0.090	−0.127	0.385	−0.148	0.306
Percentage difference between length of foot and footwear^d^ (%)	0.069	0.642	0.048	0.745	0.157	0.286	−0.058	0.694
Percentage difference between width of foot and footwear^d^ (%)	0.338	0.019	0.437	0.002	0.353^c^	0.014	0.064	0.664

^a^Parent-reported Oxford Ankle Foot Questionnaire for Children (OxAFQ-C) domain scores were based on *n* = 49 due to missing data.

^b^Pearson’s product moment correlation (*r*).

^c^Spearman’s rank-order correlation (ρ).

^d^Correlation analyses were based on *n* = 48 due to missing data.

No associations were found between Arch Index and any of the OxAFQ-C domain scores (Table 2). Mean differences and 95% confidence intervals (CI) for the associations between dichotomous scaled independent variables and the OxAFQ-C Physical, School and play, Emotional, and Footwear domain scores are shown in Table 3. No statistically significant differences were found between sexes with any OxAFQ-C domain scores. Participants with hallux valgus had significantly reduced OxAFQ-C School and play domain scores (mean difference −19.3%, 95% CI −37.7 to −1.0, *p* = 0.040). Participants with lesser toe deformities had significantly reduced OxAFQ-C Emotional domain scores (mean difference −11.5%, 95% CI −29.0 to 6.0, *p* = 0.047) (Table 3).

**Table 3:** **Univariate associations (differences between groups) between dichotomous scaled variables and OxAFQ-C domain scores**

Participant characteristics	Physical^a,b^		School and play^a^		Emotional^a^		Footwear	
	Mean difference (95% CI)	*p*value	Mean difference (95% CI)	*p*value	Mean difference (95% CI)	*p*value^c^	Mean difference (95% CI)	*p*value^c^
Sex^d^	−2.0 (−14.4 to 10.5)	0.754	−1.5 (−12.1 to 9.0)	0.771	−5.2 (−13.1 to 2.7)	0.350	6.9 (−8.8 to 22.6)	0.299
Hallux valgus^e^	−19.1 (−41.1 to 2.8)	0.062	−19.3 (−37.7 to −1.0)	0.040	−10.9 (−37.8 to 15.9)	0.133	−25.0(−50.1 to 0.1)	0.123
Lesser toe deformity^e^	−10.9 (−25.9 to 16.0)	0.297	−13.9 (−30.8 to 3.0)	0.106	−11.5 (−29.0 to 6.0)	0.047	−19.9 (−43.3 to 3.6)	0.199

^a^Parent-reported Oxford Ankle Foot Questionnaire for Children (OxAFQ-C) domain scores were based on *n* = 49 due to missing data.

^b^Analysis performed on transformed data. Results presented in antilog.

^c^The *p* values for the univariate association between dichotomous scaled variables and the OxAFQ-C Emotional domain and Footwear item scores were determined using the Mann–Whitney U Test for each variable.

^d^Condition: male minus female.

^e^Condition: presence minus absence.

Measures of the difference between the length of the participant’s foot and footwear were not significantly associated with any OxAFQ-C domain scores. However, the percentage difference between the width of the participant’s foot and footwear was significantly positively associated with three OxAFQ-C domain scores (Physical: *r* = 0.34, *p* = 0.019; School and play: *r* = 0.44, *p* = 0.002, Emotional: ρ =0.35, *p* = 0.014 respectively). These results show that increasing severity of ill-fitting footwear (narrowness) is associated with lower OxAFQ-C Physical, School and play and Emotional domain scores. There were no significant associations between footwear fit (length or width) with the OxAFQ-C Footwear domain scores (Table 2).

### Multivariate associations between foot structure and footwear fit with OxAFQ-C domain scores

Multivariate associations between foot structure and the fit of footwear with the OxAFQ-C domain scores are shown in Tables 4, 5, 6 and 7. The values that defined the lower median for the variable ill-fitting footwear were as follows: length (shoe 14.3% shorter to 10.8% longer than foot), width (shoe 26.5% to 4.9% smaller than foot).

**Table 4:** **Multivariate linear regression analyses with OxAFQ-C Physical domain scores as the dependent variable** ^**a,b**^

	OxAFQ-C Physical domain score^c^	
Independent variables	*R*^*2*^change	*p*value for*R*^*2*^change
Foot structure		
Arch Index	0.032	0.212
Hallux valgus (presence)	0.066	0.070
Lesser toe deformity (presence)	0.014	0.409
Footwear fit^d^		
Length (ill-fitting/lower median)^e^	0.013	0.431
Width (ill-fitting/lower median)^e^	0.099	0.027

^a^Adjusted for sex, age and body mass index.

^b^Parent-reported Oxford Ankle Foot Questionnaire for Children (OxAFQ-C) domain scores were based on *n* = 49 due to missing data.

^c^Analyses performed on transformed OxAFQ-C Physical domain scores.

^d^Linear regression analyses performed for footwear fit were based on *n* = 48 due to missing data.

^e^Ill-fitting: representing the greatest disparity between the participant’s foot and footwear length and width dimensions; expressed as a percentage. The values that defined the lower median (ill-fitting) were as follows: length (shoe 14.3% shorter to 10.8% longer than foot), width (shoe 26.5% to 4.9% narrower than foot).

**Table 5:** **Multivariate linear regression analyses with OxAFQ-C School and play domain scores as the dependent variable** ^**a,b**^

	OxAFQ-C School and play domain score	
Independent variables	*R*^*2*^change	*p*value for*R*^*2*^change
Foot structure		
Arch Index	0.038	0.174
Hallux valgus (presence)	0.080	0.046
Lesser toe deformity (presence)	0.037	0.179
Footwear fit^c^		
Length (ill-fitting/lower median)^d^	0.030	0.232
Width (ill-fitting/lower median)^d^	0.137	0.009

^a^Adjusted for sex, age and body mass index.

^b^Parent-reported Oxford Ankle Foot Questionnaire for Children (OxAFQ-C) domain scores were based on *n* = 49 due to missing data.

^c^Linear regression analyses performed for footwear fit were based on *n* = 48 due to missing data.

^d^Ill-fitting: representing the greatest disparity between the participant’s foot and footwear length and width dimensions; expressed as a percentage. The values that defined the lower median (ill-fitting) were as follows: length (shoe 14.3% shorter to 10.8% longer than foot), width (shoe 26.5% to 4.9% narrower than foot).

**Table 6:** **Multivariate logistic regression analyses with OxAFQ-C Emotional domain scores as the dependent variable** ^**a,b**^

	OxAFQ-C Emotional domain score^c^		
Independent variables	OR (95% CI)^d^	Change in Nagelkerke*R*^*2*^	*p*value
Foot structure			
Arch Index^e^	1.06 (0.97 to 1.16)	0.174	0.217
Hallux valgus (presence)	10.48 (0.76 to 144.17)	0.112	0.121
Lesser toe deformity (presence)	3.92 (0.33 to 46.75)	0.128	0.279
Footwear fit^f^			
Length (ill-fitting/lower median)^g^	3.78 (0.88 to 16.16)	0.199	0.073
Width (ill-fitting/lower median)^g^	5.11 (1.26 to 20.70)	0.135	0.022

^a^Adjusted for sex, age and body mass index.

^b^Parent-reported Oxford Ankle Foot Questionnaire for Children (OxAFQ-C) domain scores were based on *n* = 49 due to missing data.

^c^OxAFQ-C Emotional domain scores were dichotomised where the lowest quartile was classified as ‘disability’ and the upper three quartiles were classified as ‘no disability’.

^d^OR: Odds ratio.

^e^Arch Index multiplied by hundred.

^f^Logistic regression analyses performed for footwear fit were based on *n* = 48 due to missing data.

^g^Ill-fitting: representing the greatest disparity between the participant’s foot and footwear length and width dimensions; expressed as a percentage. The values that defined the lower median (ill-fitting) were as follows: length (shoe 14.3% shorter to 10.8% longer than foot), width (shoe 26.5% to 4.9% narrower than foot).

**Table 7:** **Multivariate logistic regression analyses with OxAFQ-C Footwear domain scores as the dependent variable** ^**a**^

	OxAFQ-C Footwear^b^domain score		
Independent variables	OR (95% CI)^c^	Change in Nagelkerke*R*^*2*^	*p*value
Foot structure			
Arch Index^d^	1.03 (0.89 to 1.18)	0.004	0.712
Hallux valgus (presence)	3.08 (0.23 to 41.79)	0.012	0.399
Lesser toe deformity (presence)	2.72 (0.17 to 43.62)	0.014	0.480
Footwear fit^e^			
Length (ill-fitting/lower median)^f^	0.23 (0.02 to 2.70)	0.061	0.240
Width (ill-fitting/lower median)^f^	0.89 (0.11 to 7.15)	0.042	0.915

^a^Adjusted for sex, age and body mass index.

^b^OxAFQ-C Footwear domain scores were dichotomised where the lowest quartile (item responses: always, very often) was classified as ‘disability’ and the upper three quartiles (item responses: never, rarely and sometimes) were classified as ‘no disability’.

^c^OR: Odds ratio.

^d^Arch Index multiplied by hundred.

^e^Logistic regression analyses performed for footwear fit were based on *n* = 48 due to missing data.

^f^Ill-fitting: representing the greatest disparity between the participant’s foot and footwear length and width dimensions; expressed as a percentage. The values that defined the lower median (ill-fitting) were as follows: length (shoe 14.3% shorter to 10.8% longer than foot), width (shoe 26.5% to 4.9% narrower than foot).

#### OxAFQ-C Physical domain

There was no significant association between foot structure and the OxAFQ-C Physical domain scores (Table 4). The use of footwear with an inappropriate length was not associated with the OxAFQ-C Physical domain scores. However, the use of footwear that was an inappropriate width was significantly associated with the OxAFQ-C Physical domain scores (in adjusted analyses, *R*^*2*^ change = 0.099, *p* = 0.027) (Table 4).

#### OxAFQ-C School and play domain

Arch Index, lesser toe deformities and the use of footwear with an inappropriate length were not associated with the OxAFQ-C School and play domain scores (Table 5). The presence of hallux valgus was significantly associated with the OxAFQ-C School and play domain scores (in adjusted analyses, *R*^*2*^ change = 0.080, *p* = 0.046). The use of footwear that was an inappropriate width was significantly associated with the OxAFQ-C School and play domain scores (in adjusted analyses, *R*^*2*^ change = 0.137, *p* = 0.009) (Table 5).

#### OxAFQ-C Emotional domain

The use of footwear that was an inappropriate width was significantly associated with the OxAFQ-C Emotional domain scores (in adjusted analyses OR 5.11, 95% CI 1.26 to 20.70, change in Nagelkerke *R*^*2*^ = 0.135, *p =* 0.022) (Table 6). In adjusted analyses, age, sex, body mass index and the presence of lesser toe deformities were not significantly associated with the OxAFQ-C Emotional domain scores (Table 6).

#### OxAFQ-C Footwear domain

None of the foot or footwear fit characteristics were significantly associated with the OxAFQ-C Footwear domain scores (Table 7).

## Discussion

Foot problems are the most frequently reported musculoskeletal disorders in children and adolescents with DS [12]. Foot-related characteristics such as flat feet, hallux valgus and ill-fitting footwear are common. However, until now, no studies have specifically investigated the association between foot structure and footwear fit with foot-specific disability in children and adolescents with DS. We found that the presence of hallux valgus was significantly associated with foot-specific disability during school and play activities. However, foot posture (that is, the severity of flat footedness) and lesser toe deformities were not associated with foot-specific disability. In addition, we found that inappropriately fitting footwear was associated with foot-specific disability. The OxAFQ-C Physical, School and play, and Emotional domain scores were significantly associated with the use of footwear that was too narrow. In contrast, there were no associations between the length fit of footwear with foot-specific disability. There was also no association found between foot structure and footwear fit with the OxAFQ-C Footwear domain scores. This finding suggests parents’ perceptions of their child’s ability to wear their shoe of choice is not associated with their child’s foot structure or fit of the footwear.

Flat feet were prevalent in our participants, which is similar to previous studies [6,16-18,28,40,41]. We hypothesised that a flat foot posture would be associated with foot-specific disability based on previous studies showing that children with DS and flat feet have reduced gait efficiency [6,41] and impaired foot function [16]. However, our findings showed that foot posture was not associated with foot-specific disability. One explanation could be that although flat footedness is associated with changes in biomechanics [6,16,41], there are no adverse effects on their ability to walk, run or engage in school and play activities (as measured using the OxAFQ-C Physical and School and play domain scores). This is supported by the notion that a paediatric flat foot is not recognised as a medical condition in the general population and children can present with a flat foot that is largely asymptomatic [42,43].

In this study, hallux valgus was associated with foot-specific disability during school and play activities. This is not surprising given that hallux valgus has been associated with greater levels of foot-specific disability in other populations [44,45]. We did not find any associations between lesser toe deformity and foot-specific disability, in contrast to older populations where deformity is associated with impaired balance and functional capacity [32]. A possible reason could be that our participants were younger and may not have been exposed to digital deformities for long enough for any adverse consequences (such as the formation of corns and calluses) to develop [33].

We found that the use of ill-fitting footwear was associated with increased levels of foot-specific disability. Specifically, there was an association between the width of footwear relative to foot width (shoes too narrow) and increased levels of OxAFQ-C disability scores, suggesting the use of footwear that is too narrow is associated with increased levels of foot-specific disability. Our findings are broadly in agreement with studies of older populations showing increased foot pain (disability) with the wearing of ill-fitting footwear [33,46]. In contrast, there was no association with length fit of the footwear. Our strategy to dichotomise footwear into appropriate or ill-fitting categories using median values as a threshold, resulted in an inappropriate shoe length as being 14.3% shorter to 10.8% longer than foot. It is widely accepted that footwear needs to be longer than the foot to allow elongation when weight-bearing [47], so it could be argued that our cut-off may have masked any associations if they existed. However, we analysed the association between footwear length and OxAFQ-C scores using a cut-off where footwear was classified as being of inadequate length was shorter than the actual length of the foot. Again, we did not find any significant associations between footwear that was shorter than the foot with foot-specific disability. Therefore, we believe our findings are robust. An explanation as to why there was a relationship between footwear width (and not length) with foot-specific disability could pertain to the ‘typical’ foot shape of children with DS. In particular, children with DS have a wider forefoot (related to the presence of hallux valgus, a greater first intermetatarsal angle or a flat foot posture) compared to children without DS [16]. Hence, children with DS are more likely to wear shoes of an inadequate width as a result of their wider forefoot.

There are limitations to the current study. First, we were limited to using the parent version of the OxAFQ-C because of anticipated difficulties in getting children and adolescents with DS to independently complete the questionnaire. Although previous work has shown that the OxAFQ-C parent-reported domain scores show moderate agreement with the child-reported domain scores, the agreement is not perfect [34]. In addition, although the OxAFQ-C has been shown to demonstrate satisfactory construct validity overall, the validity does vary across domains and is also influenced by parent or child completion. For example, the validity of the OxAFQ-C School and play domain is greater using child- than parent-reported scores [34]. Second, this is a cross-sectional study, so we cannot confirm the relationships identified in this study are causal. Third, we recruited participants from a Victorian (Australia) state cohort. Future research is required to confirm these findings in larger cohorts of children with DS to improve generalisability. Fourth, caution needs to be exercised when interpreting the study’s findings in respect to analyses involving foot structure, as the measures of the severity of hallux valgus and presence of lesser toe deformity have not been validated in child and adolescent populations. Fifth, hallux valgus and inadequate footwear width were only responsible for between 10% and 14% of the variance in the OxAFQ-C domain scores; there are likely to be several other factors that were not investigated that may be associated with foot-specific disability in children with DS. In this regard, the OxAFQ-C was designed to assess disability associated with foot and/or ankle conditions, however our study only used foot conditions. Finally, lower limb muscle hypotonia and joint laxity have also been associated with muscle fatigue and gait abnormality in children with DS, similar to children with joint hypermobility syndrome [48-50]. Hence, inclusion of these variables in future analyses may lead to more predictive models.

## Conclusions

Our study showed that foot posture and lesser toe deformities were not associated with foot-specific disability in children with DS. However, hallux valgus and the use of ill-fitting footwear, specifically footwear that was too narrow, were associated with increased levels of foot-specific disability. These findings suggest that footwear education, and regular footwear assessments may be important for children and adolescents with DS, providing the associations observed in the current study prove to be causal. Further, findings from this study warrant the need for future studies to identify other factors that may be associated with foot-specific disability in children with DS. Ideally, future work should use prospective study designs, and be conducted across the lifespan of individuals with DS.
